# Bearing State Recognition Method Based on Transfer Learning Under Different Working Conditions

**DOI:** 10.3390/s20010234

**Published:** 2019-12-31

**Authors:** Ning Cao, Zhinong Jiang, Jinji Gao, Bo Cui

**Affiliations:** 1 National Defense Key Laboratory of Ministry of Education, Beijing University of Chemical Technology, Beijing 100029, China; 18811757532@163.com; 2 Diagnostic and Self-Healing Engineering Research Center, Beijing University of Chemical Technology, Beijing 100029, China; gaojinji@263.net; 3 College of Information Science and Technology, Beijing University of Chemical Technology, Beijing 100029, China; 13146696006@163.com

**Keywords:** transfer learning, multi-core balanced distribution adaptation, SAE neural networks, rolling bearing, different working condition

## Abstract

Bearing state recognition, especially under variable working conditions, has the problems of low reusability of monitoring data, low state recognition accuracy and low generalization ability of the model. The feature-based transfer learning method can solve the above problems, but it needs to rely on signal processing knowledge and expert diagnosis experience to obtain the cross-characteristics of different working conditions data in advance. Therefore, this paper proposes an improved balanced distribution adaptation (BDA), named multi-core balanced distribution adaptation (MBDA). This method constructs a weighted mixed kernel function to map different working conditions data to a unified feature space. It does not need to obtain the cross-characteristics of different working conditions data in advance, which simplifies the data processing and meet end-to-end state recognition in practical applications. At the same time, MBDA adopts the *A*–*Distance* algorithm to estimate the balance factor of the distribution and the balance factor of the kernel function, which not only effectively reduces the distribution difference between different working conditions data, but also improves efficiency. Further, feature self-learning and rolling bearing state recognition are realized by the stacked autoencoder (SAE) neural network with classification function. The experimental results show that compared with other algorithms, the proposed method effectively improves the transfer learning performance and can accurately identify the bearing state under different working conditions.

## 1. Introduction

Modern industrial production technology makes great contributions to improving productivity, reducing losses, saving natural resources and human resources, reducing the scrap rate, and ensuring product quality. As a key piece of equipment, rotary machinery is widely applied in important engineering fields, such as power, electric power, chemical, metallurgy, mining and machinery manufacturing. Once large-scale mechanical equipment fails, it will cause huge economic losses and even cause different degrees of casualties. As a key component of rotating machinery, rolling bearings play an important role in ensuring safe and efficient operation of the machine. The working condition of the rolling bearing not only affects the operation of the machine itself, but also the subsequent production. According to statistics, in all rotating machinery faults, bearing failure accounts for about 30% [1]. Therefore, it is of great significance for the continuous production system to realize the state recognition of rolling bearings. The rapid development of signal analysis and processing technology, computer technology and network technology, and artificial intelligence provides technical support for state recognition and fault diagnosis of rolling bearings.

Early bearing state detection and fault diagnosis methods have used the vibration mechanism of rolling bearings and signal analysis and processing techniques to extract features, and have then utilized expert diagnostic experience to achieve bearing fault diagnosis and state recognition [2,3,4,5,6,7]. These methods laid the foundation for the development of bearing diagnostics. However, these methods rely too much on feature extraction and expert experience. In recent years, machine learning and deep learning have achieved good results in the fields of image recognition and natural language process. More and more scholars have applied them to the state recognition of rolling bearings [8,9,10,11]. As a special machine learning, deep learning has the characteristics of nonlinearity, multi-layer and adaptability. Therefore, it has powerful feature extraction capabilities that can automatically extract features from original data. Fault diagnosis models of rolling bearings based on deep learning mainly include convolutional neural networks (CNN) [12,13], long-term short-term memory neural networks (LSTM) [14,15], deep belief networks (DBN) [16,17], and stacked autoencoders (SAE) [18]. Compared with other network models in state recognition, SAE networks have noise reduction filtering and feature extraction functions. In particular, the SAE network with the classification function can achieve higher state recognition accuracy in small samples, which fully reflects the powerful feature extraction function and robustness of the method. The above method achieves good results in the state recognition of the rolling bearing. In practical applications, the working environment of rolling bearings is very complicated, and the working conditions often change. Under variable working conditions, training samples and test samples do not meet the same distribution conditions. Therefore, the above method will no longer apply. How to accurately identify the bearing state under different working conditions is an urgent problem to be solved.

Since the NIPS Symposium “Learning to Learn” in 1995, transfer learning has attracted more and more attention in the field of deep learning [19]. As a cross-data, cross-model and cross-task learning method, transfer learning has achieved the classification of target data by applying knowledge learned from source data [20]. Yosinski et al. [21] first studied transfer learning of deep learning and pointed out that transfer learning can solve problems such as less target data and uneven distribution of data. The feature-based transfer learning method is one of the important methods for transfer learning, mainly including transfer component analysis (TCA) [22], joint distribution adaptation (JDA) [23] and balanced distribution adaptation (BDA) [24]. In [25], the variational mode decomposition (VMD) and semi-supervised transfer component analysis (SSTCA) were proposed to achieve a bearing state classification under variable conditions. In [26], the subspace alignment (SA) was proposed to minimize the distribution difference of the domain data. In [27], a least squares support vector machine (LSSVM) transfer learning strategy based on auxiliary data was used for bearing fault diagnosis under different working conditions. The above bearing state recognition methods based on transfer learning have achieved good results, but require feature transformation or feature selection of bearing data.

Inspired by the above research, this paper uses the BDA method to transfer the vibration characteristics of rolling bearings under variable conditions. However, BDA has the following two problems: (1) The balance factor μ needs to be obtained by searching in the experiment; and (2) it needs to obtain some cross-characteristics of different domain data in advance. In order to solve the above problems, this paper proposes the MBDA method by introducing mixed kernel functions, which can extract richer features of the data without feature transformation. At the same time, the balance factor of the kernel function γ is introduced, which directly affects the effect of mixed kernel functions on data mapping. This paper adopts the A−Distance algorithm to calculate the balance factor of the distribution μ and the balance factor of the kernel function γ, which effectively minimize the distribution divergence between different domain data. Then SAE neural network is used to realize the feature self-learning and state recognition of the rolling bearing.

This paper is organized as follows. Section 2 introduces the basic principle of the BDA algorithm and SAE neural network, and gives the definition of our new algorithm with the aim to ensure that it is understandable. Section 3 gives a flow chart of the bearing diagnostic algorithm for the proposed method. We performed experiments to show the good performance of the proposed method in the bearing state identification in Section 4, and a conclusion is presented in Section 5.

## 2. Basic Theory of the Methodology

### 2.1. Balanced Distribution Adaptation

As a cross-domain, cross-model and cross-task learning method, transfer learning can effectively solve the problem of different distributions of source data and target data. Assuming the original space is X and the class label is Y, the labeled source data and the unlabeled target data are $D_{S}=\left\{X_{S},Y_{S}\right\}$ and $D_{T}=\left\{X_{T},Y_{T}\right\}$, respectively, where $X_{S},X_{T}\in X$ and $Y_{S},Y_{T}\in Y$. The marginal probability distributions and conditional probability distributions of source data and target data respectively are $P\left(X_{S}\right),P\left(Y_{S}/X_{S}\right)$ and $P\left(X_{T}\right),P\left(Y_{T}/X_{T}\right)$. In practical applications, the marginal probability distributions and conditional probability distributions of source data and target data are not equal, i.e., $P\left(X_{S}\right)\neq P\left(X_{T}\right)$ and $P\left(Y_{S}/X_{S}\right)\neq P\left(Y_{T}/X_{T}\right)$. The goal of transfer learning methods is to minimize the marginal and conditional distribution discrepancy between source data and target data. BDA as a transfer learning method can adaptively minimize the marginal and conditional distribution discrepancy between domains by exploiting a balance factor μ. The formula is as follows:

(1) $$\mathrm{Distance}\;\left(X_{S},X_{T}\right)=\left(1-\mu \right)\;\left\|P\left(X_{S}\right),P\left(X_{T}\right)\right\|+\mu \;\left\|P\left(Y_{S}/X_{S}\right),P\left(Y_{T}/X_{T}\right)\right\|$$

BDA uses the maximum mean difference (MMD) to calculate the distribution difference between the two domains. The method assumes that there is a mapping function ϕ, which satisfies $P\left(\phi \left(X_{S}\right)\right)\approx P\left(\phi \left(X_{T}\right)\right)$ and $P\left(Y_{S}/\phi \left(X_{S}\right)\right)\approx P\left(Y_{T}/\phi \left(X_{T}\right)\right)$. Therefore, Equation (1) can be represented as:

(2) $$\mathrm{Distance}\left(X_{S},X_{T}\right)=\left(1-\mu \right){\left\|\frac{1}{n_{s}}\sum_{i=1}^{n_{s}}\phi \left(x_{S_{i}}\right)-\frac{1}{n_{t}}\sum_{j=1}^{n_{t}}\phi \left(x_{T_{j}}\right)\right\|}_{H}^{2}+\mu \sum_{c=1}^{C}{\left\|\frac{1}{n_{s}{}^{\left(c\right)}}\sum_{x_{i}\in D_{S}{}^{\left(c\right)}}^{}\phi \left(x_{i}\right)-\frac{1}{n_{t}{}^{\left(c\right)}}\sum_{x_{j}\in D_{T}{}^{\left(c\right)}}^{}\phi \left(x_{j}\right)\right\|}_{H}^{2}$$

where $n_{s}$ and $n_{t}$ are the number of samples of source data and target data, respectively, and C is the number of categories.

By further taking advantage of matrix tricks and regularization, Equation (2) can be represented as:

(3) $$\begin{array}{l} \min tr\left(A^{T}X\left(\left(1-\mu \right)M_{0}+\mu \sum_{c=1}^{C}M_{c}\right)X^{T}A\right)+\lambda {\left\|A\right\|}_{F}^{2}\\ S.T\;A^{T}XHX^{T}A=I,\;0\leq \mu \leq 1 \end{array}$$

where the first term represents the marginal and conditional distribution divergences between the two domains, A denotes the transformation matrix, and λ is the regularization parameter. The constraints $A^{T}XHX^{T}A=I$ ensure that the transformed data $A{\;}^{T}X$ maintain the important properties of $X_{S}$ and $X_{T}$. $I_{n_{s}+n_{t}}\in R^{\left(n_{s}+n_{t})\times (n_{t}+n_{s}\right)}$ is the identity matrix, and $H=I_{n_{s}+n_{t}}-(1/n_{s}+n_{t})11^{T}$ is the central matrix. μ∈[0,1] is estimated by searching in the experiment. The calculation formula for each element of the $M_{0}$ and $M_{c}$ is as follows:

(4) $${\left(M_{0}\right)}_{ij}=\left\{ \begin{array}{ll} \frac{1}{n_{s}^{2}}, & x_{i},x_{j}\in D_{s}\\ \frac{1}{n_{t}^{2}}, & x_{i},x_{j}\in D_{T}\\ -\frac{1}{n_{s}n_{t}}, & \;\mathrm{otherwise} \end{array}\right.$$

(5) $${\left(M_{c}\right)}_{ij}=\left\{ \begin{array}{l} \frac{1}{n_{s}^{\left(c\right)}n_{s}^{\left(c\right)}},\;x_{i},x_{j}\in D_{s}{}^{\left(c\right)}\\ \frac{1}{n_{t}^{\left(c\right)}n_{t}^{\left(c\right)}},\;x_{i},x_{j}\in D_{T}{}^{\left(c\right)}\\ -\frac{1}{n_{s}^{\left(c\right)}n_{t}^{\left(c\right)}},\left\{ \begin{array}{l} x_{i}\in D_{s}{}^{\left(c\right)},x_{j}\in D_{T}{}^{\left(c\right)}\\ x_{i}\in D_{T}{}^{\left(c\right)},x_{j}\in D_{s}{}^{\left(c\right)} \end{array}\right.\\ 0,\;\mathrm{otherwise} \end{array}\right.$$

The above non-convex optimization problem is transformed into the trace optimization problem by the Lagrange multiplier method, and the specific process will not be described.

### 2.2. Multi-Core Balanced Distribution Adaptation

BDA has the following two problems: (1) it is inefficient to get the balance factor μ; and (2) it needs to obtain some cross-characteristics of different domain data in advance. In order to solve the above problems, this paper proposes the MDBA method. The kernel function plays an important role in the BDA. The effect of a single kernel function in transfer learning is not ideal. Weighted mixed kernel functions combine the advantages of different kernel functions, but add a new parameter γ. The formula for weighted mixed kernel functions is as follows:

(6) $$\begin{array}{ll} K\prime (x_{i},x_{j}) & =\gamma K_{RBF}(x_{i},x_{j})+(1-\gamma )K_{Ploy}(x_{i},x_{j})\\ & =\gamma \exp\left(-\frac{{\left\|x_{i}-x_{j}\right\|}^{2}}{2\sigma^{2}}\right)+(1-\gamma ){\left[\left(x_{i}\cdot x_{j}\right)+1\right]}^{d} \end{array}$$

where $K_{RBF}$ and $K_{Ploy}$ are the radial basis function (RBF) and ploynomial kernel (Ploy) function respectively, and γ∈[0,1] controls the weight of the two kernel function. The formula of $K_{RBF}$ and $K_{Ploy}$ is follows:

(7) $$K_{RBF}(x_{i},x_{j})=\exp\left(-\frac{{\left\|x_{i}-x_{j}\right\|}^{2}}{2\sigma^{2}}\right)$$

(8) $$K_{Poly}(x_{i},x_{j})={\left[\left(x_{i}\cdot x_{j}\right)+1\right]}^{d}$$

MBDA adopts A−Distance to empirically estimate γ and the specific process is as follows:

(1) Use SVM to train two-classifiers h to distinguish source data from target data and obtain the loss value err(h);
(2) Calculate A−Distance between source data and target data, and the formula is as follows:
  (9) $$A\left(X_{S},X_{T}\right)=2\left(1-2err\left(h\right)\right)$$
(3) Calculate the balance factor γ, and the formula is as follows:
  (10) $$\gamma =\frac{A_{Ploy}\left(X_{S}^{\prime },X_{T}^{\prime }\right)}{A_{RBF}\left(X_{S}^{\prime },X_{T}^{\prime }\right)+A_{Poly}\left(X_{S}^{\prime },X_{T}^{\prime }\right)}$$
  where $X_{S}^{\prime }$ and $X_{T}^{\prime }$ respectively represent source data and target data after kernel mapping. $A_{RBF}\left(X_{S}^{\prime },X_{T}^{\prime }\right)$ and $A_{Ploy}\left(X_{S}^{\prime },X_{T}^{\prime }\right)$ represent the values of A−Distance. The larger the value is, the greater the difference between source data and target data after the kernel mapping, so the weight of the corresponding kernel function is smaller, and vice versa.

The balance factor μ plays an important role in minimizing the marginal and conditional distribution discrepancy between the domains. BDA evaluates the μ by searching its values in experiments, but it is not an effective solution. In order to effectively adjust the marginal and conditional distribution of the importance on different tasks, we estimate the balance factor μ by adopting A−Distance, and its formula is as follows:

(11) $$\mu =\frac{A_{\mathrm{Marginal}}\left(X_{S}^{\prime },X_{T}^{\prime }\right)}{A_{\mathrm{Marginal}}\left(X_{S}^{\prime },X_{T}^{\prime }\right)+A_{\mathrm{Conditional}}\left(X_{S}^{\prime },X_{T}^{\prime }\right)}$$

where $A_{\mathrm{Marginal}}\left(X_{S}^{\prime },X_{T}^{\prime }\right)$ represents the A−Distance value of the marginal probability distribution for source and target domains, and $A_{\mathrm{Conditional}}\left(X_{S}^{\prime },X_{T}^{\prime }\right)$ represents the A−Distance value of the conditional probability distributions for the source and target domains. When μ→0, it means that there is a big difference between source data and target data. Therefore, the marginal distribution adaptation is more important, and vice versa.

### 2.3. Stacked Autoencoder Neural Network

#### 2.3.1. Autoencoder

**Figure 1:**
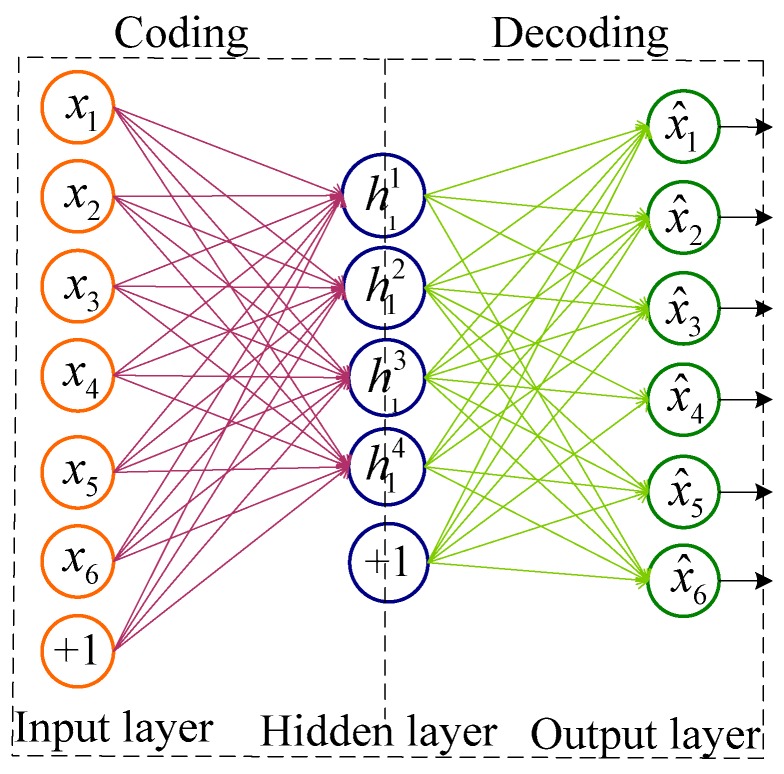
Autoencoder structure diagram.

As one of the classic models of neural networks, the autoencoder consists of two stages of encoding and decoding. Its structure is shown in Figure 1. The autoencoder converts the original data of the high-dimensional space into the coding vector of the low-dimensional space by encoding, and then reconstructs the coding vector into the original data by decoding. The specific implementation process is as follows:

- Coding phase: the information is transmitted from front to back.
  (12) $$\begin{array}{l} h_{1}=f\left(z_{1}\right)\\ z_{1}=w_{1}x_{1}+b_{1} \end{array}$$
  where, assuming the input layer is $X=\left\{x_{1},x_{2},\ldots x_{n}\right\}$, the subscript in the formula indicates that there are n training samples, $w_{1}$ and $b_{1}$ are the weight and bias of the encoding layer, respectively, and f(∗) is the excitation function, which is usually a sigmoid or tanh function.
- Decoding phase: the information is transmitted from the back to the front:
  (13) $$\begin{array}{l} {\hat{x}}_{1}=f\left(z_{2}\right)\\ z_{2}=w_{2}h_{1}+b_{2} \end{array}$$
  where ${\hat{x}}_{1}$ is the output value of the decoding layer, $w_{2}$ and $b_{2}$ are the weights and bias of the decoding layer, respectively, and $w_{2}=w_{1}^{T}$.

As can be seen from the above process, the autoencoder belongs to unsupervised training. The training process of the autoencoder is to find the network parameters to minimize the reconstruction error on the training set D. The reconstruction error is generally the quadratic cost function, and the expression is as follows:

(14) $$\begin{array}{l} J_{AE}=\sum_{x\in D}L\left({\hat{x}}_{1},x_{1}\right)\\ L\left({\hat{x}}_{1},x_{1}\right)=\frac{1}{N}{\left\|{\hat{x}}_{1}-x_{1}\right\|}^{2} \end{array}$$

where $\hat{X}$ is the predicted value, *N* is the number of samples, and *L* is the reconstructed error function. When the reconstruction error is small enough, the compressed feature vectors of the encoding layer retain most of the information of the original data.

#### 2.3.2. Stack Autoencoder Neural Network

**Figure 2:**
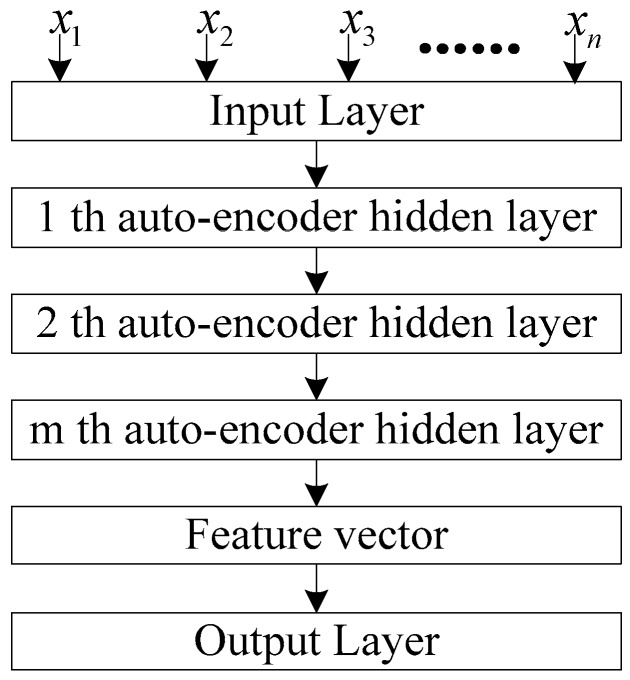
Stacked Autoencoder Neural Network.

The SAE neural network is a deep neural network composed of multiple autoencoders. The basic principle is that the output of the previous autoencoder is used as the input of the latter autoencoder. Its structure is shown in Figure 2. Compared with the autoencoder network, the SAE neural network is more expressive and can extract more abundant features from original data. The encoding process of an m-layer SAE neural network is as follows:

(15) $$\begin{array}{l} a^{\left(l\right)}=f\left(z^{\left(l\right)}\right)\\ z^{\left(l+1\right)}=w^{\left(l,1\right)}a^{\left(l\right)}+b^{\left(l,1\right)} \end{array}$$

where $a^{\left(l\right)}$ is the output value of the lth encoding layer; $w^{\left(l,1\right)},b^{\left(l,1\right)}$ is the weight and bias of the lth encoding layer; and $z^{\left(l\right)}$ and $z^{\left(l+1\right)}$ are the input value of the lth layer and the l+1th layer, respectively. The SAE neural network transmits information from the back to the front at the decoding process:

(16) $$\begin{array}{l} a^{\left(m+l\right)}=f\left(z^{\left(m+l\right)}\right)\\ z^{\left(m+l+1\right)}=w^{\left(m-l,2\right)}a^{\left(m+l\right)}+b^{\left(m-l,2\right)} \end{array}$$

where $a^{\left(m\right)}$ is the activation value of the deepest hidden unit, and $w^{\left(n-l,2\right)},b^{\left(n-l,2\right)}$ are the weight and bias of the decoding layer. The SAE network structure is as follows:

The training process of the SAE neural network with classification function includes two stages: model pre-training and model fine-tuning:

- Model pre-training. The SAE neural network is constructed and the network model parameters are initialized through the unsupervised layer-by-layer training mode. Through pre-training, all hidden layers are obtained, and the features learned at each layer represent different levels of data characteristics.
- Model fine-tuning. Add a classification layer at the top of the SAE network and fine-tune the pre-training parameters to implement the classification function. Fine-tuning training takes all the layers of the SAE neural network as a whole model to train. At each iterative training, each parameter of the model is optimally adjusted. Therefore, fine-tuning training can improve the performance of SAE deep neural networks.

In this paper, the quadratic cost function and the cross-entropy cost function are used as the objective function of the first and second stages. The quadratic cost function is elaborated in Section 2.3.1, and the cross-entropy cost function is as follows:

(17) $$z_{i}^{j}=\frac{e^{h_{\theta ,j}\left(x_{i}\right)}}{\sum_{j=1}^{C}e^{h_{\theta ,j}\left(x_{i}\right)}}$$

(18) $$J\left[\theta \right]=-\frac{1}{N}\sum_{i=1}^{N}\sum_{j=1}^{C}\;\left\{\;y_{i}=j\;\right\}\;\log\left(z_{i}^{j}\right)$$

where $z_{{}_{i}}^{j}$ is the predicted probability that the $x_{i}$ belongs to category j, $h_{\theta ,j}\left(x_{i}\right)$ represents the jth value of the output vector, and θ is neural network parameter. The SAE neural network can automatically extract features and has powerful feature expression capabilities. In fault diagnosis, the SAE neural network has the functions of noise reduction filtering and feature extraction.

## 3. Bearing State Recognition Method and Process under Different Working Conditions

**Figure 3:**
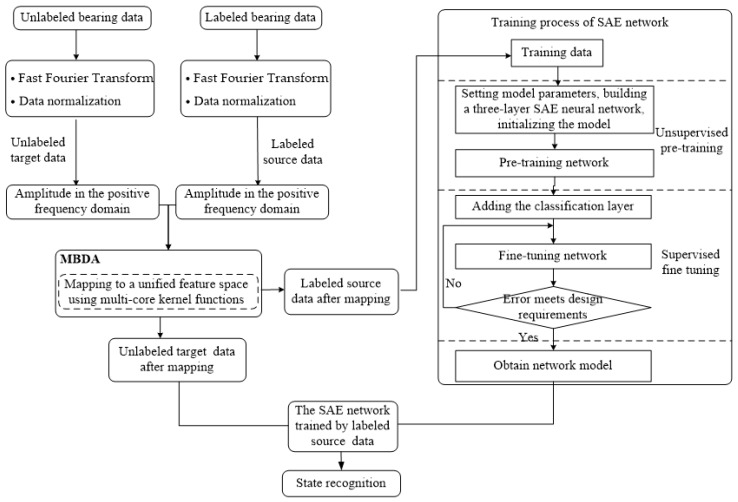
Flowchart of the bearing state recognition method under different working conditions.

The algorithm flow of the bearing state recognition method based on transfer learning under different working conditions is shown in Figure 3.

The specific implementation process is as follows:

1. Calculate the spectrum of labeled bearing data and unlabeled bearing data, and normalize the amplitude to the range of [0, 1]. Because the signal’s spectral amplitude is symmetrical about the origin, the positive frequency part is used as feature vectors. This not only ensures that information is not lost, but also reduces the number of calculations. The positive frequency domain amplitude of labeled bearing data is used as labeled source data, and the positive frequency domain amplitude of unlabeled bearing data is used as unlabeled target data.
2. Map labeled source data and unlabeled target data in (1) to the same feature space by using the MBDA algorithm.
3. The training process of SAE is also a feature self-learning process, which can further extract features. The training process of SAE includes two parts: unsupervised pre-training and supervised fine tuning. Unsupervised pre-training is used to initialize network parameters, and supervised fine-tuning implements classification by adding a classification layer on top of the network. Labeled source data after spatial mapping in (2) are used as training samples to train the model, and finally the training model is obtained. Unlabeled source data after spatial mapping in (2) are input into the model, and the rolling bearing state recognition results are obtained.

## 4. Experimental Verification

### 4.1. Experimental Data

The experimental data are from the bearing data center of the Case Western Reserve University Laboratory. In this experiment, the SKF6205 drive end bearing vibration data is used as experimental data. Four different rotational speeds and different motor loads represent different working conditions A, B, C and D. Each working station includes four states: normal state (NS), internal raceway fault (IF), external raceway fault (OF) and ball fault (BF). The detailed information of the experimental data is shown in Table 1.

In Table 1, the fault diameter of IF, OF and BF indicates the diameter of a bearing inner raceway fault, outer raceway fault and ball fault. This paper constructs data sets under single and multiple working conditions, as shown in Table 2. 

Where A(T)-B(S) indicates that the source data (training data) is from working condition B, and the target data (test data) is from working condition A; A(T)-BC(S) indicates that source data (training data) is from working condition BC, and target data (test data) is from working condition A; AB(T)-CD(S) indicates that source data (training data) is from working condition CD, and target data (test data) is from working condition AB; A(T)-BCD(S) indicates that source data (training data) is from working condition BCD, and target data (test data) is from working condition A.

### 4.2. Model Performance Analysis

**Figure 4:**
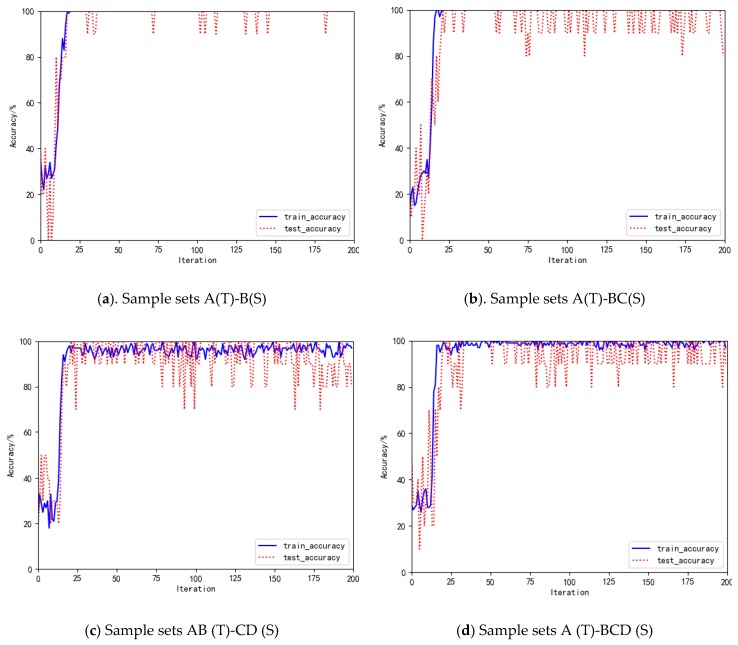
Bearing state recognition accuracy curve under working conditions A(T)-B(S), A(T)-BC(S), AB (T)-CD (S) and A (T)-BCD (S), respectively.

In the experiment, source data (training data) and target data (test data) are selected from single or multiple working conditions data sets. Taking A(T)-B(S) as an example, labeled source data B(S) is used to train the SAE neural network, and unlabeled target data A(T) is input into the model to obtain the bearing state. Training samples after fast Fourier transform (FFT) are normalized, and the amplitude of the positive frequency part is taken as a feature vector to train the SAE network. In the experiment, the normalized positive frequency part of the vibration data is used as the feature vector. This not only ensures that information is not lost, but also reduces the number of calculations. Generally, at neural network structure, the higher the number of network layers, the stronger the network expression ability. However, when the number of network layers is too large, it is difficult to train the model. After the previous experiments, this paper uses a three-layer SAE network model, the structure of the network is set to 64-32-16, bath_size is set to 100, and the number of iterations is 200. In the experiment, the normalized positive frequency part of the vibration data is used as the feature vector. This not only ensures that information is not lost, but also reduces the number of calculations. Under the four working conditions A(T)-B(S), A(T)-BC(S), AB (T)-CD (S) and A (T)-BCD (S), the curves of bearing state recognition accuracy of this method are shown in Figure 4.

Figure 4a–d are graphs showing the state recognition accuracy of test data (target data) and training data (source data) under four working conditions, respectively. The trend of the accuracy of training data (source data) and test data (target data) is synchronized. Therefore, there is no over-fitting. In the case of single/single conditions A (T)-B (S), the state recognition accuracy of the test data almost reaches 100%. In the case of single/multiple conditions A (T)-BC(S) and A (T)-BCD(S), the state recognition accuracy of the test data is 98.50% and 96.86%, respectively. In the case of multiple/multiple conditions AB (T)-CD (S), the state recognition accuracy of the test data reaches almost 90.50%. The experimental results show that the MBDA-SAE method can obtain higher state recognition accuracy under variable working conditions.

### 4.3. Analysis and Comparison of MBDA and other Algorithms

In order to prove the advantages of the MBDA method, this paper introduces traditional transfer learning methods such as the TCA, JDA and BDA methods. The results after different transfer learning methods and SAE networks under variable working conditions are shown in Table 3. As can be seen from the above table, the state recognition accuracy of the MBDA-SAE method is higher than that of the TCA-SAE, JDA-SAE, and BDA-SAE methods. The reason is that the multi-core kernel function has a good advantage in dealing with the imbalance between source data and target data. JDA-SAE and BDA-SAE methods take the difference of marginal distribution and conditional distribution as objective functions, so their state accuracy is higher than the TCA-SAE method. Compared to the JDA-SAE method, the BDA method adds a balance factor obtained by searching in experiments to balance the marginal distribution and conditional distribution. The BDA-SAE method achieves higher state recognition accuracy at the expense of efficiency.

## 5. Conclusions

In this paper, we propose a rolling bearing state recognition method based on a WDBA-SAE neural network under different working condition data. The following conclusions have been obtained through experiments:

(1) This method depends on BDA theory, and constructs a weighted mixed kernel function to map different working condition data to a unified feature space, which effectively minimizes the distribution divergence between different working conditions data. The MDBA method does not need to obtain the cross-characteristics of different working conditions data in advance, which simplifies data processing.
(2) This paper adopts the A−Distance algorithm to calculate the balance factor of the distribution and the balance factor of the kernel function. It can adaptively balance the importance of the marginal and conditional distribution and the importance of different kernel functions, and improve efficiency.
(3) The MDBA method was compared to other transfer learning methods, such as TCA, JDA and BDA. In the case of a single/single condition A (T)-B (S), the accuracy of the bearing state recognition methods based on the JDA-SAE, BDA-SAE and MDBA-SAE methods reached more than 90%. However, the diagnostic accuracy based on the TCA-SAE method is 75%. In the case of multiple/multiple conditions AB (T)-CD (S), the state recognition accuracy of the method proposed in this paper reaches more than 90%. However, the accuracy of other methods is less than 80%. Therefore, the advantages of this method are more obvious under multiple/multiple conditions. Experiments showed that the MDBA method can better recognize the unknown state of rolling bearings under variable working conditions.

The follow-up work of this article is as follows:

(1) During the deep neural network training process, multiple experiments are required to determine better hyperparameters (such as the number of network layers, the number of neurons, the number of iterations, etc.), and then the setting of the hyperparameters will be studied;
(2) The features extracted from the multi-layer network feature space will be visualized;
(3) This article only studies bearing-related faults, and subsequent studies will distinguish other faults, such as unbalanced loads and broken rotor bars.

## Figures and Tables

**Table 1:** Vibration signal parameter table for experimental data.

Different Working Conditions	RPM (r/min)	Motor Load (W)	Fault Diameter of IF, OF and BF (mm)	Fs (kHz)	Number of Samples
A	1730	2.25	0.1778	12	1500
B	1750	1.5	0.3356		1500
C	1772	0.75	0.5334		1500
D	1797	0	0.7112		1500

**Table 2:** Sample sets composition of rolling bearings under different working conditions.

Sample Sets	Source Data	Target Data	Source Data Sample Number	Target Data Sample Number
Single/single conditions	B	A	1500	1500
Single/multiple conditions	BC	A	3000	1500
Multiple/multiple conditions	CD	AB	3000	3000
Single/multiple conditions	BCD	A	4500	1500

**Table 3:** Bearing state recognition accuracy under different methods (%).

Different Methods/Sample Sets	A(T)-B(S)	A(T)-BC(S)	AB(T)-CD(S)	A(T)-BCD(S)	Average Accuracy
TCA-SAE	75.00	69.00	62.00	54.00	65.00
JDA-SAE	92.00	77.00	69.52	69.00	76.88
BDA-SAE	96.99	88.00	83.10	77.00	86.27
MBDA-SAE	100.00	98.50	96.86	90.50	96.47

## References

[1] Sun H., He Z., Zi Y., Yuan J., Wang X., Chen J., He S. (2014). Multiwavelet transform and its applications in mechanical fault diagnosis—A review. Mech. Syst. Signal Process..

[2] Tse P.W., Peng Y.H., Yam R. (2001). Wavelet analysis and envelope detection for rolling element bearing fault diagnosis - their effectiveness and flexibilities. J. Vib. Acoust..

[3] Žvokelj M., Zupan S., Prebil I. (2016). EEMD-based multiscale ICA method for slewing bearing fault detection and diagnosis. J. Sound Vib..

[4] Ma B., Jiang Z.N. (2004). Envelope analysis based on Hilbert transform and its application in rolling bearing fault diagnosis. J. B. Univ. Chem. Technol..

[5] Xu Y., Meng Z., Zhao G. (2014). Study on compound fault diagnosis of rolling bearing based on dual-tree complex wavelet transform. Chin. J. Sci. Instrum..

[6] Ren Z., Zhou S., Chunhui E., Gong M., Li B., Wen B. (2015). Crack fault diagnosis of rotor systems using wavelet transforms. Comput. Electr. Eng..

[7] Chen X.H., Cheng G., Shan X.L., Hu X., Guo Q., Liu H.G. (2015). Research of weak fault feature information extraction of planetary gear based on ensemble empirical mode decomposition and adaptive stochastic resonance. Measurement.

[8] Guo L., Gao H., Zhang Y., Huang H. (2016). Research on Bearing State Recognition Based on Deep Learning Theory. J. Vib. Shock.

[9] Kilundu B., Letot C., Dehombreux P., Chiementin X. (2008). Early Detection of Bearing Damage by Means of Decision Trees. IFAC Proc. Vol..

[10] Wu S.D., Wu C.W., Wu P.H., Ding J.J., Wang C.C. (2012). Bearing fault diagnosis based on multiscale permutation entropy and support vector machine. Entropy.

[11] Satish B., Sarma N.D.R. A fuzzy bp approach for diagnosis and prognosis of bearing faults in induction motors. Proceedings of the IEEE Power Engineering Society General Meeting (PESGM).

[12] Li H., Zhang H., Qin X.R., Sun Y.T. (2018). Fault diagnosis method for rolling bearings based on short-time Fourier transform and convolution neural network. J. Vib. Shock.

[13] Wang F., Jiang H., Shao H. (2017). An adaptive deep convolutional neural network for rolling bearing fault diagnosis. Meas. Sci. Technol..

[14] Wang F., Liu X., Deng G., Yu X., Li H., Han Q. (2019). Remaining Life Prediction Method for Rolling Bearing Based on the Long Short-Term Memory Network. Neural. Process Lett..

[15] Hinchi A.Z., Tkiouat M. (2018). Rolling element bearing remaining useful life estimation based on a convolutional long-short-term memory network. Procedia. Comput. Sci..

[16] Shao H., Jiang H., Wang F., Wang Y. (2016). Rolling bearing fault diagnosis using adaptive deep belief network with dual-tree complex wavelet packet. ISA Trans..

[17] Shao H., Jiang H., Zhang X., Niu M. (2015). Rolling bearing fault diagnosis using an optimization deep belief network. Meas. Sci. Technol..

[18] Chen R.X., Yang X., Yang L.X. (2017). Fault severity diagnosis method for rolling bearings based on a stacked sparse denoising auto-encoder. J. Vib. Shock.

[19] Duan L., Tsang I.W., Xu D. (2012). Domain transfer multiple kernel learning. IEEE Trans. Pattern Anal. Mach. Intell..

[20] Pan S.J., Yang Q. (2009). A survey on transfer learning. IEEE Trans. Knowl. Data Eng..

[21] Yosinski J., Clune J., Bengio Y., Lipson H. (2014). How transferable are features in deep neural networks?. Adv. Neural Inf. Process. Syst..

[22] Pan S.J., Tsang I.W., Kwok J.T., Yang Q. (2011). Domain Adaptation via Transfer Component Analysis. IEEE Trans. Neural Netw..

[23] Wang J., Chen Y., Hao S., Feng W., Shen Z. Balanced Distribution Adaptation for Transfer Learning. Proceedings of the 2017 IEEE International Conference on Data Mining (ICDM).

[24] Long M., Wang J., Ding G., Sun J., Yu P.S. Transfer Feature Learning with Joint Distribution Adaptation. Proceedings of the 2013 IEEE International Conference on Computer Vision.

[25] Kang S.Q., Hu M.W., Wang Y.J., Xie J., Mikulovich V.I. (2019). Fault Diagnosis Method of a Rolling Bearing Under Variable Working Conditions Based on Feature Transfer Learning. Proc. CSEE.

[26] Fernando B., Habrard A., Sebban M., Tuytelaars T. Unsupervised visual domain adaptation using subspace alignment. Proceedings of the IEEE international conference on computer vision (ICCV 2013).

[27] Chen C., Shen F., Yan R. (2017). Enhanced least squares support vector machine-based transfer learning strategy for bearing fault diagnosis. Chin. J. Sci. Instrum..

